# War-related and psychological predictors of experiential and physiological fear of war in Germany: findings from a nationally representative survey

**DOI:** 10.1186/s12889-026-29548-7

**Published:** 2026-09-22

**Authors:** Katja Linde, Julia Treml, Jana Reinhardt, Jörg M. Fegert, Emily Sitarski, Elmar Brähler, Anette Kersting

**Affiliations:** 1 Department of Psychosomatic Medicine and Psychotherapy, University of Leipzig, University Medical Center, Semmelweisstraße 10, Leipzig, 04103 Germany; 2 Department of Child and Adolescent Psychiatry, Psychosomatics and Psychotherapy, Ulm University Hospital, Steinhövelstraße 5, Ulm, 89075 Germany; 3 German Center for Mental Health (DZPG), partner site Ulm, Steinhövelstraße 5, Ulm, 89075 Germany; 4 Competence Center Public Child Mental Health, partner site Ulm, Steinhövelstraße 5, Ulm, 89075 Germany; 5 Clinic and Polyclinic for Psychosomatic Medicine and Psychotherapy, Rostock University Medical Center, Gehlsheimer Straße 20, Rostock, 18147 Germany; 6 Department for Psychosomatic Medicine and Psychotherapy, University Medical Center, Johannes Gutenberg University of Mainz, Untere Zahlbacher Str. 8, Mainz, 55131 Germany

**Keywords:** Fear of war, War anxiety, Experiential fear, Physiological fear, Geopolitical threat, Armed conflict, Public mental health, Psychological vulnerability, Interpersonal trust, Emotion regulation

## Abstract

**Background:**

Fear of war has emerged as a relevant psychosocial phenomenon in Europe, yet little is known about factors associated with its experiential and physiological dimensions in representative populations. This study examined sociodemographic, war-related, and psychological predictors of experiential and physiological fear of war in Germany.

**Method:**

Data were drawn from a nationally representative cross-sectional survey of German-speaking individuals aged 16 years and older (*N* = 2,530). Fear of war was assessed using the Fear of War Scale (FOWARS). Hierarchical multiple regression analyses were conducted separately for experiential and physiological fear of war, entering sociodemographic, psychological, and war-related variables in three theory-driven blocks.

**Results:**

Participants reported moderate levels of experiential fear of war (*M* = 3.54, *SD* = 0.99), whereas physiological fear was lower on average (*M* = 2.24, *SD* = 1.12). The final models explained 9% of the variance in experiential fear of war and 11% of the variance in physiological fear of war. War-related salience emerged as a key correlate of experiential fear, particularly when participants referred to a specific war, a global/world war, or nuclear war. In contrast, physiological fear showed clearer associations with indicators of psychological vulnerability, including childhood adversity, lower interpersonal trust, and emotion regulation variables. Overall, the findings supported differential predictor patterns across experiential and physiological fear. Women and older individuals reported higher fear levels. Sensitivity analyses using an ordinal salience variable supported the robustness of the findings.

**Conclusions:**

Fear of war appears to reflect an interplay between war-related salience and individual vulnerability. Distinguishing experiential and physiological fear responses may improve understanding of psychosocial reactions to geopolitical crises and contribute to public mental health monitoring and risk communication.

**Supplementary Information:**

The online version contains supplementary material available at https://doi.org/10.1186/s12889-026-29548-7.

## Highlights

• This study is among the first to examine psychological and war-related predictors of experiential and physiological fear of war in a representative population sample.

• War-related salience and psychological vulnerability showed different patterns of associations with experiential and physiological fear.

• Findings highlight the importance of considering both current threat salience and individual vulnerability when examining psychosocial responses to geopolitical crises.

**Supplementary Information:**

The online version contains supplementary material available at https://doi.org/10.1186/s12889-026-29548-7.

## Background

 In recent years, geopolitical tensions in Europe and beyond have intensified considerably. Russia’s full-scale invasion of Ukraine in February 2022 marked a major shift in the European security landscape, alongside escalating conflicts in the Middle East and renewed debates about nuclear threats. Wars are relevant not only because of their immediate physical consequences, but also because of their broader psychosocial impact. Forced displacement is increasing worldwide, and conflict exposure is associated with a substantial burden of mental disorders, particularly depression, anxiety, and posttraumatic stress disorder, in affected populations [1, 2]. This broader psychosocial burden is also reflected in recent studies conducted during the Russian–Ukrainian war, which found substantial psychological distress among Ukrainian university populations and associations between coping strategies and mental health among Ukrainian and Polish students [3, 4]. Recent reports further indicate rising conflict intensity and a growing proportion of the global population living in conflict-affected settings [5, 6].

Fear of war is not limited to directly exposed populations, but may also occur in countries without immediate war exposure. Research on collective stressors suggests that responses to large-scale threats are shaped not only by objective proximity, but also by media exposure and subjective threat appraisal. Distress following the September 11 attacks, the Boston Marathon bombing, and the COVID-19 pandemic has been linked to media exposure and threat-related communication processes [7–10]. Consistent with the Social Amplification of Risk Framework, societal communication may intensify perceived risk beyond direct exposure [11]. In Europe, well-being declined on the day of Russia’s invasion of Ukraine and was lower on days when the war was more salient in social media environments [12]. Together, these findings suggest that geopolitical crises may affect mental health beyond directly exposed populations.

From a theoretical perspective, fear is not a one-dimensional phenomenon. Classical and contemporary models distinguish subjective experience from physiological threat responses [13, 14]. This distinction is reflected in multidimensional approaches to fear of war. Against this background, war-related distress has been described using terms such as fear of war and war anxiety. In the present study, the term *fear of war* is used to refer to fear of being personally affected by war, either directly or indirectly [15]. Many population-based studies have relied on single-item indicators or broader anxiety measures, limiting the differentiation of fear components [16, 17]. To address this limitation, the Fear of War Scale (FOWARS) was developed as a multidimensional measure comprising experiential and physiological components of fear of war [15]. Initial studies supported its two-factor structure and psychometric adequacy [15, 18]. However, representative studies examining predictors of both dimensions within a common explanatory framework remain lacking.

Psychological vulnerability and regulatory processes may help explain individual differences in fear of war. According to transactional stress models, emotional responses depend on threat appraisal and perceived coping resources [19]. Childhood adversity has been linked to heightened stress reactivity and enduring neurobiological alterations, suggesting increased vulnerability to later stressors [20–22]. Higher childhood adversity may therefore be associated with higher fear of war, particularly with physiological fear responses. Interpersonal trust may function as a protective social resource in complex and uncertain contexts. Higher trust has been linked to perceived stability and safety when individuals rely on external information [23, 24]. Lower trust may therefore be associated with higher fear of war under conditions of unpredictability and limited personal control. Emotion regulation strategies represent another relevant domain because they influence how individuals respond to difficult-to-control stressors. Two widely studied strategies are cognitive reappraisal, which involves changing the interpretation of a situation, and expressive suppression, which involves inhibiting emotional expression [25, 26]. Reappraisal is often associated with more adaptive emotional outcomes, whereas suppression has more often been linked to higher distress and reduced well-being [27]. In prolonged and largely uncontrollable situations such as war, these strategies may therefore be associated with fear responses.

War-related factors such as salience and proximity are also plausible correlates of fear of war. War-related salience refers to the subjective relevance and mental availability of war-related threat, including thinking about specific conflicts or severe escalation scenarios. Persistent war-related thinking has been linked to higher distress [12, 28]. Perceived proximity may increase the personal relevance of threat through close social ties to affected regions or prior experiences in conflict-affected settings. Research on stress appraisal and risk perception suggests that perceived threat and personal relevance are important determinants of emotional responses [19, 29]. However, the incremental contribution of war-related salience and proximity relative to individual vulnerability has not been systematically examined in representative samples.

Sociodemographic characteristics may shape vulnerability to societal threat. Gender differences are well documented in anxiety research [30]. German population data further suggest associations with partnership status and parenthood, whereas findings regarding age are less consistent [17]. Fear of war has also been associated with poorer mental health, including anxiety, depressive symptoms, and stress, highlighting its clinical and public health relevance [15, 16, 31].

Despite converging evidence from stress, trauma, and risk-perception research, few studies have jointly examined sociodemographic, war-related, and psychological predictors of fear of war. It also remains unclear whether these domains relate differently to experiential versus physiological fear responses. Therefore, this study addressed the following overarching research question: To what extent are psychological vulnerability, emotion regulation, and war-related salience and proximity associated with experiential and physiological fear of war in a nationally representative German sample? Based on theoretical models of stress appraisal and prior research on war-related fear, trauma, and psychological vulnerability, the following hypotheses were formulated.

### Hypotheses

- H1 (Psychological vulnerability and regulation): Greater childhood adversity, lower interpersonal trust, lower cognitive reappraisal, and higher expressive suppression were expected to be associated with higher fear of war.
- H2 (War-related salience and proximity): Greater war-related salience and greater proximity to war were expected to be associated with higher fear of war.
- H3 (Differential associations across dimensions). Predictors were expected to show different patterns of association with experiential and physiological fear of war, potentially reflecting distinct cognitive–emotional and physiological response patterns.

## Methods

### Procedures

The analyses are based on a nationally representative cross-sectional survey of German-speaking individuals conducted by USUMA (Berlin) between October 10, 2024, and February 15, 2025. Sampling followed a stratified, multistage random design based on ADM procedures. In a first step, sampling points were selected across Germany according to regional and urban–rural stratification. Subsequently, households were identified using a standardized random-route procedure. Within each household, one eligible participant was randomly selected using the Kish selection grid [32].

Trained interviewers conducted face-to-face interviews followed by self-report questionnaires. Inclusion criteria were age ≥ 16 years and sufficient German language proficiency. All participants provided informed consent, with parental consent obtained for minors. The study was approved by the local ethics committee (reference 316/24-ek) and conducted in accordance with the Declaration of Helsinki.

### Participants

A total of 6,499 target individuals were contacted by 171 trained interviewers. Nonresponse resulted primarily from household refusal (39.0%), unsuccessful contact (9.7%), and individual refusal (9.5%), whereas other reasons accounted for 1.1% or less. In total, 2,552 interviews were completed. After excluding 22 cases with incomplete or invalid data, the final sample comprised 2,530 participants (response rate: 38.9%).

### Measures

Sociodemographic information was measured with a self-constructed questionnaire and included age, gender, partnership status, children in the household (< 16 years), education, and employment status.

#### Fear of war

Fear of war was assessed using the German version of the Fear of War Scale (FOWARS; [15]), a 13-item self-report measure of fear of being personally affected by war. Items are rated on a 5-point Likert scale from 1 (strongly disagree) to 5 (strongly agree), with higher scores indicating greater fear of war. The scale comprises two correlated subscales assessing experiential and physiological fear of war. The experiential dimension captures cognitive and emotional aspects of war-related fear (e.g., “I am concerned about the serious consequences of the war”) and the physiological dimension assesses bodily stress reactions (e.g., “My palms sweat when I think the war will reach us too”). Previous studies demonstrated good reliability and validity across different language versions [15, 18].

The German version of the FOWARS was translated from the original English version using established forward–backward translation procedures involving multiple bilingual native speakers. Discrepancies between translated versions were discussed within the research team until consensus was achieved. In the present sample, internal consistencies were good to excellent (experiential dimension: *α* = 0.92, ω = 0.91; physiological dimension: *α* = 0.95, ω = 0.95).

#### Psychological vulnerability and emotion regulation

Childhood adversity, interpersonal trust, and emotion regulation were assessed as indicators of psychological vulnerability and regulatory processes.

#### Childhood adversity

Childhood adversity was assessed using the German version of the Childhood Trauma Questionnaire (CTQ; [33, 34]), which assesses emotional abuse, physical abuse, sexual abuse, emotional neglect, and physical neglect. Items are rated on a 5-point Likert scale ranging from 1 (not at all) to 5 (very often), with higher scores indicating greater levels of childhood adversity.

For the present analyses, a mean score was computed to index overall childhood adversity. This approach is consistent with prior research supporting the reliability and validity of the German Childhood Trauma Questionnaire and the use of total scores as a general vulnerability indicator for mental health outcomes [33, 35]. In the present sample, internal consistency of the total mean score was good (*α* = 0.91, ω = 0.92).

#### Generalized interpersonal trust

Interpersonal trust was assessed using the 3-item short scale of interpersonal trust (KUSIV-3; Kurzskala Interpersonelles Vertrauen; [36, 37]), a brief self-report measure of generalized trust in others. Items are rated on a 5-point Likert scale ranging from 1 (strongly disagree) to 5 (strongly agree), with higher scores indicating greater interpersonal trust.

Items were averaged to form a composite mean score. Previous research has demonstrated satisfactory reliability and validity of the KUSIV3 in population-based samples [36]. In the present sample, internal consistency was modest to acceptable (*α* = 0.63, ω = 0.72).

#### Emotion regulation strategies

Emotion regulation strategies were assessed using the 10-item Emotion Regulation Questionnaire (ERQ; [26, 38, 39]), measuring cognitive reappraisal and expressive suppression. Items are rated on a 7-point Likert scale ranging from 1 (strongly disagree) to 7 (strongly agree), with higher scores indicating more frequent use of the respective strategy. Previous research has demonstrated good reliability and validity of the ERQ [26]. Mean scores were calculated for both subscales. Internal consistencies in the present sample were acceptable to good (reappraisal: *α* = 0.88, ω = 0.88; suppression: *α* = 0.84, ω = 0.84).

#### War-related salience and proximity variables

War-related salience and proximity were assessed using self-report items capturing the subjective relevance and personal proximity of war-related experiences.

Participants were instructed to respond to the fear of war items with regard to war in general. To assess whether responses were nevertheless anchored in specific conflict representations, participants were asked whether they had a particular war in mind while completing the items. Participants answering affirmatively were additionally asked to name the war they had predominantly thought about in an open-ended format. Open-ended responses were subsequently coded into five mutually exclusive categories reflecting qualitatively different forms of war-related salience: no specific war (0), one specific war (1), multiple wars (2), global/world war (3), and nuclear war (4). Categories were derived using inductive content coding and subsequently formalized in a predefined coding manual. To assess inter-coder reliability, a second coder, blinded to the original classifications and FOWARS scores, independently coded a random sample of 250 open-ended responses using the same coding manual. Responses that could not be meaningfully assigned to one of the predefined categories by at least one coder were excluded from the reliability analysis (*n* = 7), resulting in 243 valid cases. Inter-coder agreement was 98.8%, with an unweighted Cohen’s κ of 0.97. For the main regression analyses, the salience categories were represented using four dummy-coded variables (specific war, multiple wars, global/world war, nuclear war), with no specific war serving as the reference category. In addition, for sensitivity analyses, the five-category salience variable was treated as an ordinal representation of perceived threat intensity ranging from 0 (no specific war) to 4 (nuclear war threat).

War-related proximity was assessed using two dichotomous indicators. The first assessed whether participants had close others (e.g., family members or friends) from countries currently affected by war. The second assessed whether participants had previously spent extended time in a country currently affected by war. Both variables were coded as 0 = no and 1 = yes.

### Statistical analyses

Statistical analyses were conducted using IBM SPSS Statistics version 29. The significance level was set to *α* = 0.05.

Missing data at the item level were low (0-1.3%); therefore, no item-level imputation was applied. Scale scores were computed as mean values when at least 75% of items were available, resulting in scale-level missing data of 0.2–0.6%. Listwise deletion was used for the regression analyses, yielding analytic samples of *N* = 2,408 (95.2% of total sample) for experiential fear and *N* = 2,410 (95.3% of total sample) for physiological fear.

Descriptive statistics and bivariate correlations were computed to examine variable distributions and associations.

To examine predictors of fear of war, theory-driven hierarchical multiple linear regression analyses were conducted separately for the experiential and physiological dimensions. Differences in predictor patterns between experiential and physiological fear were interpreted descriptively; corresponding regression coefficients were not formally compared. Predictors were entered in three blocks. In the first block, sociodemographic variables were included as covariates (age, gender, education, employment status, partnership status, and children in the household). Dichotomous variables were coded 0/1, and categorical variables (education, employment) were dummy-coded, with medium education and being employed as reference categories. In the second block, psychological variables were entered, including childhood adversity (CTQ mean score), interpersonal trust (KUSIV3 mean score), and emotion regulation (ERQ), operationalized as mean scores for cognitive reappraisal and expressive suppression. War-related salience was represented by four dummy-coded variables reflecting references to one specific war, multiple wars, global/world war, and nuclear war, with no specific war serving as the reference category. War-related proximity was represented by two dichotomous indicators assessing whether participants had close others from war-affected regions and whether they had previously spent extended time in a war-affected country.

Regression results are reported as standardized regression coefficients (*β*), which were used to describe the magnitude of associations for individual predictors in the final models. Changes in explained variance (Δ*R*²) were examined to evaluate the incremental contribution of each block. To quantify the magnitude of the incremental contribution of the psychological and war-related predictor blocks, Cohen’s *f*² [40] was calculated by comparing each extended model with the preceding reduced model, using *f*² = (*R*²_full − *R*²_reduced)/(1 − *R*²_full).

Assumptions of linear regression were examined prior to interpretation of the results. Linearity and homoscedasticity were assessed via residual plots, and normality via histograms and Q–Q plots. Multicollinearity was evaluated using tolerance (< 0.10) and VIF (> 10). Independence of residuals was assessed with the Durbin–Watson statistic (≈ 2). Influential cases were screened using standardized residuals (|z| > 3), leverage, and Cook’s distance.

### Sensitivity analyses

To examine the robustness of the findings, regression models were rerun using alternative operationalizations of war-related salience, including an ordinal representation of the salience categories ranging from no specific war to nuclear war threat. In addition, one-way analyses of variance (ANOVAs) with Games–Howell post hoc tests were conducted to compare fear levels across salience categories.

## Results

### Descriptive analyses

A total of 2,530 participants completed the survey (54.0% female; *M_*age = 49.42 years, *SD* = 17.72, range = 16–92 years). The majority were living with a partner (59.5%), had a medium level of education (59.5%), and were employed (61.8%). When asked about the war they had in mind while responding to the FOWARS items, 52.5% of participants referred to one specific war. Among these, the Russia–Ukraine war was most frequently mentioned (86.4%), followed by the Israel–Gaza war (9.5%). In contrast, 33.9% did not name a specific conflict. Smaller proportions referred to multiple wars (5.7%), a global or world war threat (6.3%), or nuclear war (1.7%). In addition, 8.5% reported that close others were currently affected by war, and 3.0% reported an extended stay in a war-affected country. Further sample characteristics are presented in Table 1.

**Table 1:** Demographic and war-related characteristics of the total sample (*N* = 2,530)

Age, M (SD)	49.42 (17.72)
Gender, *n* (%)	
Female	1365 (54.0)
Male	1163 (46.0)
Partnership, *n* (%)	
Yes	1485 (59.5)
No	1012 (40.5)
Children under 16 in household, *n* (%)	
Yes	480 (19.0)
No	2048 (81.0)
Education, *n* (%)^a^	
Low	700 (27.7)
Medium	1503 (59.5)
High	288 (11.4)
Currently attending school	35 (1.4)
Employment status, *n* (%)^b^	
Unemployed	96 (3.8)
Employed	1564 (61.8)
Outside labour force^b^	853 (33.9)
Type of war referenced, *n* (%)	
No specific war	848 (33.9)
One specific war	1313 (52.5)
Multiple different wars	142 (5.7)
Global/World war	158 (6.3)
Nuclear war	42 (1.7)
Close others currently affected by war, *n* (%)	
Yes	215 (8.5)
No	2315 (91.5)
Extended stay in a war-affected country, *n* (%)	
Yes	76 (3.0)
No	2454 (97.0)

Percentages are based on available data. Missing values were observed for gender (*n* = 2), partnership (*n* = 33), children under 16 in household (*n* = 2), education (*n* = 4), employment status (*n* = 17), and type of war (*n* = 27)

^ᵃ^Education level (low = left school without certificate or lower secondary certificate; medium = intermediate secondary certificate, polytechnic school certificate, technical school certificate, or higher education entrance qualification; high = completed university degree). ᵇEmployment status (e.g., voluntary service/parental leave, retired, not employed, vocational training, school, or higher education)

Mean experiential fear was slightly above the scale midpoint, whereas mean physiological fear was below the midpoint. Childhood adversity levels were low, whereas interpersonal trust and cognitive reappraisal were slightly above the midpoint of their respective scales. Expressive suppression was approximately at the midpoint. Overall, bivariate associations among sociodemographic, war-related, and psychosocial variables were predominantly small (Table 2).

**Table 2:** Descriptive statistics and zero-order correlations among study variables

		M	SD	1	2	3	4	5	6	7	8	9
1	Experiential dimension (FOWARS subscale mean score)	3.54	0.99	--								
2	Physiological dimension (FOWARS subscale mean score)	2.24	1.12	0.50**	--							
3	Age	49.42	17.72	0.15**	0.11**	--						
4	Female (0 = male, 1 = female)	0.54	0.50	0.12**	0.13**	0.02	--					
5	In partnership/married (0 = no, 1 = yes)	0.59	0.49	0.02	− 0.08**	0.02	− 0.03	--				
6	Children under 16 in household (0 = no, 1 = yes)	0.19	0.39	− 0.00	− 0.01	− 0.30**	0.07**	0.21**	--			
7	Low education (ref: medium)	0.28	0.45	− 0.00	0.03	0.25**	− 0.04*	− 0.10**	− 0.07**	--		
8	High education (ref: medium)	0.11	0.32	0.00	− 0.07**	0.02	− 0.04*	0.03	0.01	− 0.22**	--	
9	Currently in school (ref: medium)	0.01	0.12	− 0.02	0.00	− 0.22**	− 0.03	− 0.14**	0.05*	− 0.07**	− 0.04*	--
10	Unemployed (ref: employed)	0.04	0.19	− 0.05*	− 0.03	− 0.07**	− 0.04*	− 0.08**	− 0.03	0.13**	− 0.03	− 0.02
11	Outside labour force (ref: employed)	0.34	0.47	0.07**	0.11**	0.39**	0.04*	− 0.18**	− 0.19**	0.19**	− 0.04*	0.16**
12	Specific war (ref: no specific war)	0.52	0.50	0.07**	− 0.09**	− 0.04*	0.00	0.07**	− 0.01	− 0.03*	0.03	− 0.00
13	Multiple wars (ref: no specific war)	0.06	0.23	0.05*	− 0.01	0.04*	0.01	0.01	− 0.01	− 0.02	0.04*	0.00
14	Global war (ref: no specific war)	0.06	0.24	0.09**	0.16**	0.02	0.03	− 0.04*	0.02	0.01	− 0.06**	− 0.02
15	Nuclear war (ref = no specific war)	0.02	0.13	0.08**	0.11**	0.01	0.00	− 0.03	− 0.02	0.02	− 0.01	0.06**
16	Close others affected by war (0 = no, 1 = yes)	0.08	0.28	0.08**	0.00	− 0.11**	− 0.00	0.05**	0.06**	− 0.05**	0.11**	0.05**
17	Extended stay in a country currently affected by war (0 = no, 1 = yes)	0.03	0.17	0.04*	− 0.03	− 0.01	− 0.03	0.03	0.04*	− 0.05**	0.10**	− 0.00
18	Childhood adversity (CTQ mean score)	1.43	0.46	0.01	0.10**	0.09**	− 0.01	− 0.03	− 0.01	0.18**	− 0.05**	− 0.04*
19	Interpersonal trust (KUSIV3 mean score)	3.20	0.79	− 0.01	− 0.12**	0.02	0.04*	0.03	0.00	− 0.13**	0.14**	0.05**
20	Cognitive reappraisal (ERQ subscale mean score)	4.54	1.26	0.09**	0.07**	− 0.02	0.05**	0.02	0.04*	− 0.05**	0.03	0.06**
21	Expressive suppression (ERQ subscale mean score)	3.95	1.41	0.06**	0.09**	0.08**	− 0.12**	− 0.06**	− 0.02	0.12**	− 0.04*	− 0.03

	10	11	12	13	14	15	16	17	18	19	20	21
1												
2												
3												
4												
5												
6												
7												
8												
9												
10	--											
11	− 0.14**	--										
12	− 0.01	− 0.04*	--									
13	− 0.01	0.04*	− 0.26**	--								
14	− 0.00	0.03*	− 0.27**	− 0.06**	--							
15	− 0.03	0.05**	− 0.14**	− 0.03	− 0.03*	--						
16	0.02	− 0.03*	0.07**	0.02	− 0.04*	− 0.03	--					
17	0.00	− 0.00	0.06**	− 0.02	− 0.04*	− 0.01	0.41**	--				
18	0.16**	0.06**	− 0.09**	− 0.01	0.05**	0.02	0.05**	− 0.01	--			
19	− 0.13**	0.01	0.04	0.02	− 0.06**	0.01	0.03	0.03	− 0.25**	--		
20	− 0.11**	− 0.04*	0.02	− 0.03	0.01	− 0.04*	0.04*	0.04*	− 0.14**	0.15**	--	
21	0.06**	− 0.01	− 0.05**	− 0.03	0.03	0.04*	0.03	0.07**	0.14**	− 0.13**	0.18**	--

*CTQ*  Childhood Trauma Questionnaire, *KUSIV3*  Interpersonal Trust Short Scale, *ERQ*  Emotion Regulation Questionnaire. *p <.05, **p <.01

Assumptions for multiple linear regression were adequately met for both outcome variables. No substantial deviations from linearity, homoscedasticity, normality, multicollinearity (all VIFs ≤ 1.38), or independence of errors (Durbin–Watson = 1.83 and 1.76) were observed, and no influential cases affected the results. Model fit statistics are presented in Table  3 and regression coefficients in Table 4. Standardized regression coefficients reported below refer to the final hierarchical regression models (Model 3). Across both final models, statistically significant standardized coefficients ranged from |*β*| = 0.04 to 0.17 and indicated predominantly small individual associations.

**Table 3:** Model fit of hierarchical regression models predicting experiential and physiological fear of war

Outcome				*R*	*R*²	Adjusted *R*²	ΔR²	Incremental Cohen’s f²
Experiential fear of war								
		Model 1: Sociodemographic variables		0.20	0.04	0.04	-	-
		Model 2: + Psychological variables		0.22	0.05	0.04	0.01***	0.01
		Model 3: + War-related variables		0.31	0.09	0.09	0.04***	0.05
Physiological fear of war								
			Model 1: Sociodemographic variables	0.21	0.04	0.04	-	-
			Model 2: + Psychological variables	0.27	0.07	0.07	0.03***	0.03
			Model 3: + War-related variables	0.33	0.11	0.10	0.04***	0.04

*R*  multiple correlation coefficient, *R*² proportion of explained variance, Adjusted *R*² variance explained adjusted for the number of predictors, Δ*R*² change in explained variance compared with the previous model. Cohen’s *f*² represents the incremental effect size associated with the addition of the psychological and war-related predictor blocks and was calculated from the corresponding full and reduced models. Psychological variables included childhood adversity, interpersonal trust, and emotion regulation. War-related variables included salience and proximity indicators

*** p < .001

**Table 4:** Final hierarchical regression models predicting experiential and physiological fear of war

Predictor	Experiential fear			Physiological fear		
	B	β	*p*	B	β	*p*
Sociodemographic variables						
Age	0.01	**0.17**	**< 0.001**	0.01	**0.08**	**< 0.001**
Female (ref: male)	0.23	**0.12**	**< 0.001**	0.29	**0.13**	**< 0.001**
In partnership/married (ref: no)	0.02	0.01	0.699	-0.14	**− 0.06**	**0.003**
Children under 16 in household (ref: no)	0.08	0.03	0.154	0.04	0.01	0.536
Low education (ref: medium)	-0.08	− 0.04	0.103	-0.09	− 0.03	0.113
High education (ref: medium)	-0.02	− 0.01	0.784	-0.15	**− 0.04**	**0.034**
Currently in school (ref: medium)	0.07	0.01	0.683	0.00	0.00	0.991
Unemployed (ref: employed)	-0.09	− 0.02	0.388	-0.16	− 0.03	0.174
Outside labour force (ref: employed)	0.01	0.01	0.765	0.14	**0.06**	**0.007**
Psychological variables						
Childhood adversity (CTQ)	-0.01	− 0.00	0.841	0.16	**0.07**	**0.001**
Interpersonal trust (KUSIV-3)	-0.04	− 0.03	0.119	-0.16	**− 0.11**	**< 0.001**
Cognitive reappraisal (ERQ)	0.05	**0.06**	**0.006**	0.07	**0.07**	**< 0.001**
Expressive suppression (ERQ)	0.04	**0.05**	**0.016**	0.04	**0.05**	**0.021**
War-related salience and proximit**y**						
One specific war (ref: no specific war)	0.31	**0.16**	**< 0.001**	-0.02	− 0.01	0.697
Multiple wars (ref: no specific war)	0.38	**0.09**	**< 0.001**	0.02	0.00	0.863
Global war (ref: no specific war)	0.55	**0.14**	**< 0.001**	0.65	**0.14**	**< 0.001**
Nuclear war (ref: no specific war)	0.84	**0.11**	**< 0.001**	1.02	**0.12**	**< 0.001**
Close others affected by war (ref: no)	0.33	**0.09**	**< 0.001**	0.16	0.04	0.059
Extended stay in a country currently affected by war (ref: no)	-0.07	− 0.01	0.580	-0.21	− 0.03	0.139

Values represent unstandardized regression coefficients (*B*), standardized regression coefficients (*β*), and *p* values from the final hierarchical regression models (Model 3). Sociodemographic variables were entered in Model 1, psychological variables in Model 2, and war-related salience and proximity variables in Model 3. Higher scores on continuous predictors indicate greater childhood adversity (CTQ), greater interpersonal trust (KUSIV-3), and greater use of the respective emotion regulation strategies (ERQ). Reference categories are indicated in parentheses

Bold values indicate statistically significant associations (*p* < .05)

### Predictors of experiential fear of war

For experiential fear of war, the final regression model was statistically significant, *F*(19, 2388) = 12.86, *p* < .001. Sociodemographic variables (Model 1) explained 4% of the variance. Adding psychological variables (Model 2) explained an additional 1% of the variance (Δ*R²* = 0.01, *f*² = 0.01, *p* < .001), corresponding to a very small incremental effect. The inclusion of war-related salience and proximity variables (Model 3) explained an additional 4% of the variance (Δ*R²* = 0.04, *f*² = 0.05, *p* < .001), corresponding to a small incremental effect. The final model accounted for 9% of the variance in experiential fear of war (*R²* = 0.09, adjusted *R²* = 0.09).

Among sociodemographic covariates, older age (*β* = 0.17, *p* < .001) and female gender *(β* = 0.12, *p* < .001) were associated with higher experiential fear of war, whereas other sociodemographic variables were not significant. Regarding psychological variables (hypothesis 1), both cognitive reappraisal (*β* = 0.06, *p* = .006) and expressive suppression (*β* = 0.05, *p* = .016) showed small positive associations with experiential fear of war, while childhood adversity and interpersonal trust were not significantly related. War-related salience variables (hypothesis 2) were consistently associated with experiential fear of war. Thinking about one specific war (*β* = 0.16, *p* < .001), multiple wars (*β* = 0.09, *p* < .001), global war threat (*β* = 0.14, *p* < .001), and nuclear war threat (*β* = 0.11, *p* < .001) were all associated with higher experiential fear of war. In addition, having close others from war-affected regions was positively associated with experiential fear of war (*β* = 0.09, *p* < .001), whereas prior extended stay in a war-affected country was not significant (see Table 4).

### Predictors of physiological fear of war

For physiological fear of war, the final regression model was statistically significant, *F*(19, 2390) = 15.33, *p* < .001. Sociodemographic variables (Model 1) explained 4% of the variance. Adding psychological variables (Model 2) explained an additional 3% of the variance (Δ*R*² = 0.03, *f*² = 0.03, *p* < .001), corresponding to a small incremental effect. The inclusion of war-related salience and proximity variables (Model 3) explained a further 4% of the variance (Δ*R*² = 0.04, *f*² = 0.04, *p* < .001), likewise corresponding to a small incremental effect. The final model accounted for 11% of the variance in physiological fear of war (*R*² = 0.11, adjusted *R*² = 0.10).

Among sociodemographic covariates, older age (*β* = 0.08, *p* < .001) and female gender (*β* = 0.13, *p* < .001) were associated with higher physiological fear of war. Being in a partnership (*β* = −0.06, *p* = .003) and having higher education (*β* = −0.04, *p* = .034) were related to slightly lower physiological fear, whereas being outside the labour force predicted slightly higher physiological fear (*β* = 0.06, *p* = .007). Other sociodemographic variables were not significant.

Regarding psychological variables (hypothesis 1), greater childhood adversity (*β* = 0.07, *p* = .001), lower interpersonal trust (*β* = −0.11, *p* < .001), cognitive reappraisal (*β* = 0.07, *p* < .001), and expressive suppression (*β* = 0.05, *p* = .021) were associated with higher physiological fear of war.

Among war-related variables (hypothesis 2), global war threat (*β* = 0.14, *p* < .001) and nuclear war threat (*β* = 0.12, *p* < .001) were positively associated with physiological fear of war. In contrast, thinking about one specific war or multiple wars, having close others from war-affected regions, and prior extended stay in a war-affected country were not significantly related (see Table 4).

### Differential predictor patterns across fear dimensions

As shown in Table 4, the two fear dimensions showed different descriptive predictor patterns. War-related salience variables showed a more consistent pattern of association with experiential fear, whereas childhood adversity, interpersonal trust, and emotion regulation variables showed clearer associations with physiological fear.

### Sensitivity analyses

Sensitivity analyses using an ordinal representation of war-related salience (ranging from no specific war to nuclear war threat) yielded a similar overall pattern. Higher values on the ordinal salience variable were associated with higher experiential fear (*β* = 0.18, *p* < .001) and physiological fear (*β* = 0.14, *p* < .001). Complementary one-way ANOVAs confirmed significant differences between salience conditions for experiential, *F*(4, 2483) = 23.85, *p* < .001, *η*² = 0.04, and physiological fear, *F*(4, 2485) = 25.78, *p* < .001, *η*² = 0.04.

Games–Howell post hoc tests showed that experiential fear was significantly lower in the no-specific-war group than in all other groups (*ps* < 0.001). In addition, participants referring to global or nuclear war scenarios showed higher experiential fear than those referring to one specific war (*ps* ≤ 0.001), whereas differences between the remaining conditions were not statistically significant (*ps* ≥ 0.10).

**Fig. 1:**
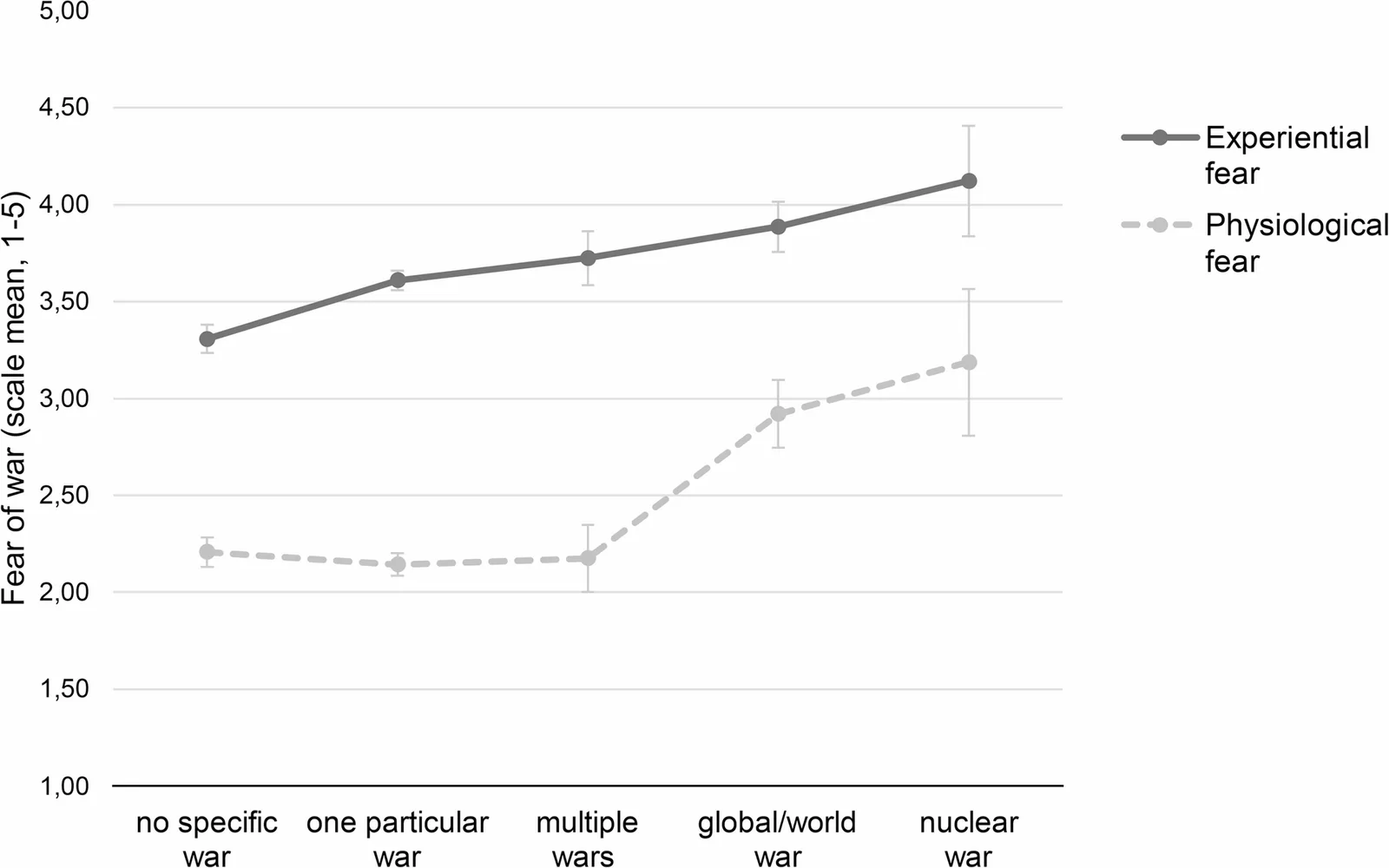
Mean experiential and physiological fear of war across war-related salience categories. Error bars represent 95% confidence intervals

In contrast, physiological fear did not differ between the no-specific-war, specific-war, and multiple-war conditions (*ps* ≥ 0.68). However, participants referring to global or nuclear war scenarios reported higher physiological fear than participants in the remaining conditions (*ps* < 0.001), with no difference between the global and nuclear war conditions (*p* = .70). Descriptive patterns are illustrated in Fig. 1, and full post hoc comparisons are reported in Additional file 1: Table S1.

## Discussion

This population-based study examined sociodemographic, war-related, and psychological predictors of experiential and physiological fear of war in Germany. Three main findings emerged. First, war-related salience emerged as a key correlate of experiential fear. Second, physiological fear showed clearer associations with vulnerability indicators such as childhood adversity and lower interpersonal trust. Third, women and older individuals reported higher levels of fear.

### Summary of main findings

Although the overall explained variance was modest (*R*² ≈ 0.09–0.11), the incremental effects of the psychological and war-related predictor blocks ranged from very small to small (*f*² = 0.01–0.05), and standardized regression coefficients ranged from |*β*| = 0.04 to 0.17, indicating predominantly small associations. In population-based psychological research, effects of this magnitude can nevertheless be meaningful, particularly when the underlying exposure affects large segments of the population [41, 42]. At the same time, the coefficients should not be interpreted as evidence of strong individual-level prediction or causality.

The modest explained variance likely reflects the multifactorial nature of war-related fear, shaped by situational developments, media dynamics, and individual differences not captured in the present dataset. Fear of war may also be sensitive to short-term contextual fluctuations that are difficult to assess in cross-sectional designs. Nevertheless, several theoretically meaningful predictors emerged. Experiential fear was more consistently associated with war-related salience, whereas physiological fear showed clearer associations with indicators of psychological and social vulnerability, including greater childhood adversity and lower interpersonal trust. Women and older individuals also reported higher fear levels. Taken together, the findings suggest that fear of war reflects an interplay between situational threat perception and individual vulnerability. This overall pattern can be interpreted by integrating the Social Amplification of Risk Framework with transactional stress theory. Societally communicated threats may become more mentally salient, while emotional responses depend on individual threat appraisal and coping resources [11, 19]. From this perspective, war-related salience may reflect the mental availability of geopolitical threat, whereas childhood adversity and interpersonal trust may reflect more enduring differences in vulnerability and perceived social safety.

### War salience and socially mediated threat processes

War-related salience emerged as the central correlate of experiential fear. When war becomes mentally salient, threat-related representations may become more accessible and intensify cognitive-emotional fear responses. This interpretation is consistent with evidence showing that responses to large-scale threats, such as pandemics, terrorism, or war-related crises, vary with salience in the broader information environment [10, 12]. Sensitivity analyses suggested that experiential fear varied across the ordinal war-related salience categories, with particularly elevated levels in participants referring to global or nuclear war scenarios. In contrast, physiological fear was elevated primarily in these global and nuclear conditions. The content of the represented threat may therefore also matter. Global and nuclear threats combine catastrophic scope, limited controllability, and potentially irreversible consequences—characteristics associated with heightened perceptions of dread risk [29]. Consistent with the relevance of nuclear threat, recent research during the Russian–Ukrainian war found nuclear anxiety to be associated with greater war-related concern as well as higher anxiety and depressive symptoms [43]. However, this interpretation remains tentative because the salience categories were derived from open-ended responses and do not constitute a validated intensity continuum.

Although not directly assessed, media-related processes may represent one pathway through which war-related salience becomes psychologically active. Repeated exposure to crisis-related media content has been associated with increased stress responses even in indirectly exposed populations, and more compulsive patterns of media consumption have additionally been linked to higher fear of war [7, 8, 44]. In Finnish university students, more time spent following the Russian–Ukrainian war via media was associated with more sleep problems and greater anxiety and depressive symptoms [45].

In addition, having close others from war-affected regions was associated with slightly higher experiential fear, whereas prior extended stays in such regions were not. This pattern suggests that social proximity may be more relevant than direct exposure, with experiential fear potentially amplified through socially mediated threat processes, such as secondary stress within close relationships [46, 47].

### Psychological vulnerability: childhood adversity and interpersonal trust

Physiological fear showed clearer associations with indicators of psychological and social vulnerability. Greater childhood adversity and lower interpersonal trust were linked to higher physiological fear, suggesting that war-related cues elicit stronger bodily threat responses in individuals with greater vulnerability. This interpretation is consistent with evidence that early-life adversity can become biologically embedded in stress-regulatory systems and increase later physiological reactivity to threat [20, 21]. The observed association is also compatible with a cumulative-vulnerability perspective, as repeated or co-occurring childhood adversities may contribute to greater allostatic burden and later stress sensitivity [48]. However, the CTQ mean score used in the present study reflects overall maltreatment severity across domains rather than the number of adverse experiences and therefore does not directly test a cumulative dose–response relationship. This pattern also aligns with theoretical models suggesting that physiological threat responses may partly differ from consciously experienced fear appraisals [14]. Accordingly, war-related cues may trigger stronger bodily arousal in more vulnerable individuals.

Interpersonal trust may function as a protective social resource, as it has been linked to perceived stability, reduced uncertainty, and lower risk perception in complex contexts [23, 49]. In situations characterized by uncertainty and limited personal control, such as war or geopolitical conflict, higher trust may buffer physiological activation, whereas lower trust may be associated with greater perceived unpredictability and risk perception [50].

### Emotion regulation and fear of war

An unexpected finding was that both cognitive reappraisal and expressive suppression were positively associated with fear of war. This contrasts with classical models that conceptualize reappraisal as relatively adaptive and suppression as less adaptive [25, 26, 51]. However, the effectiveness of emotion regulation strategies may depend on contextual factors and on the flexible selection of strategies in response to situational demands [52–54].

The positive association between cognitive reappraisal and fear should not be interpreted as evidence that reappraisal increases fear. The ERQ assesses habitual strategy use rather than the effectiveness of war-specific emotion regulation, and individuals experiencing greater fear may engage more frequently in attempts to regulate these emotions. Higher reappraisal may therefore reflect ongoing regulatory engagement rather than successful fear reduction, while suppression may similarly reflect attempts to manage heightened arousal. Taken together, these findings are consistent with a context-sensitive account in which emotion-regulation strategies are not uniformly adaptive or maladaptive. Given the cross-sectional design, however, the direction of these associations cannot be determined.

### Age and gender differences

Women reported higher levels of fear of war than men, consistent with well-established gender differences in fear and anxiety research more broadly [30, 55] as well as with recent findings on war-related fear in Germany [17]. Older age was associated with higher experiential fear and, to a lesser extent, physiological fear. This may reflect greater perceived realism of geopolitical risk or cohort effects, as older generations may have lived through the Second World War, the Cold War, or other societal threats. Age-related differences in risk perception may also contribute to heightened fear responses in older individuals [24]. However, these interpretations remain tentative, as cohort effects and historical exposure were not directly assessed.

### Differential predictor patterns and theoretical implications

The differential predictor pattern supports the usefulness of distinguishing experiential and physiological fear, consistent with the conceptual framework of the FOWARS [15]. The descriptive differences between the two fear dimensions may reflect partly distinct components of threat responding. Experiential fear primarily captures consciously accessible concern, anticipation, and emotional appraisal and may therefore be more closely linked to the conflict representation participants had in mind, whereas the physiological subscale captures subjectively perceived bodily arousal and may be more closely related to individual vulnerability. The present findings are consistent with multidimensional theories of fear and with transactional stress models emphasizing interactions between threat appraisal, vulnerability, and coping resources [13, 14, 19], as well as with evidence that subjective fear and physiological threat responses may show partly dissociable patterns [56]. This suggests that the two dimensions capture related but partly distinct aspects of fear of war that may be relevant for both research and prevention. Importantly, the physiological FOWARS subscale reflects self-reported bodily reactions rather than direct physiological measurement, and the observed differences between the two fear dimensions should be interpreted descriptively, as corresponding regression coefficients were not formally compared.

### Strengths and limitations

A key strength of the study is the use of a large nationally representative sample, allowing a population-level perspective on fear of war and its correlates. A further strength is the relatively balanced representation of women and men in the sample, as men are often underrepresented in survey research. Several limitations should be noted. First, the cross-sectional design precludes causal conclusions, and longitudinal studies are needed to clarify temporal and potentially bidirectional relationships. This is particularly relevant for war-related salience because the open-ended salience question followed the fear-of-war items, such that participants’ fear levels may have influenced the conflict scenarios subsequently retrieved or reported. Second, the explained variance was modest, suggesting that additional factors may contribute to fear of war. Future research could consider variables such as personality characteristics, intolerance of uncertainty, media exposure, and situational escalation dynamics. Third, the war-related salience variable may have captured not only the mental availability of war-related threats but also differences in perceived threat severity, particularly for global and nuclear war scenarios. Future studies should therefore assess salience and perceived threat intensity separately. Moreover, the ordinal sensitivity analysis imposed an ordering of these categories that has not been validated as an intensity continuum and should therefore be considered exploratory. Fourth, the open-ended war-related salience responses required classification into researcher-developed categories and therefore involved some interpretive judgment, although the very high inter-coder agreement observed in the present study supports the reliability of the coding. Some salience categories, particularly nuclear war, were also small, limiting the precision of category-specific estimates. Fifth, childhood adversity was assessed retrospectively by self-report and may therefore be subject to recall, social desirability, and other response biases, including potential under- or overreporting of sensitive experiences. Sixth, the brief interpersonal trust measure (KUSIV3) showed only modest internal consistency in the present sample, which may have attenuated associations with fear of war. Future studies may benefit from using more comprehensive measures of interpersonal trust. Finally, generalizability beyond Germany may be limited, and cross-cultural studies are needed to examine whether the observed patterns replicate in other sociopolitical and cultural contexts.

### Implications

The association between war-related salience and experiential fear suggests that factors increasing the mental accessibility of geopolitical threats may be relevant to fear responses. Although media exposure was not directly assessed, previous research indicates that repeated exposure to crisis-related media content may contribute to heightened stress and fear responses [7, 8, 10]. Public mental health approaches may therefore consider not only objective proximity to conflict, but also subjective war-related salience, which may be influenced by repeated or graphic media exposure. Future research could examine whether media literacy and exposure-management approaches are useful in this context. In addition, population-level monitoring may help identify periods of elevated psychosocial burden and population groups experiencing greater distress. The findings further suggest that individual vulnerability factors, including childhood trauma and lower interpersonal trust, may be relevant when examining vulnerability to heightened distress in response to war-related threats. Such monitoring may be particularly relevant during periods of heightened geopolitical instability, when fear responses can extend beyond directly exposed populations. A better understanding of factors associated with fear of war may therefore inform public mental health surveillance and risk communication during geopolitical crises.

### Conclusion

In this large nationally representative German sample, fear of war appears to reflect an interplay between war-related salience and individual vulnerability. While war-related salience was more consistently associated with experiential fear, indicators of psychological vulnerability, including childhood adversity and lower interpersonal trust, showed clearer associations with physiological fear. These findings underscore the usefulness of distinguishing experiential and physiological dimensions of fear of war and suggest partly distinct patterns of association with geopolitical threat and individual vulnerability. Given the cross-sectional design and modest explained variance, these patterns should be interpreted as associations rather than distinct causal pathways. Given the growing relevance of geopolitical crises, a better understanding of these dimensions may contribute to public mental health monitoring and risk communication.

## Supplementary Information

Supplementary Material 1: Additional file 1: Table S1. Games–Howell post hoc comparisons of experiential and physiological fear of war across war-related salience categories. The file contains post hoc comparisons between war-related salience groups for experiential and physiological fear of war.

## Data Availability

Due to data protection and privacy regulations, the datasets generated and analyzed in this study cannot be made publicly available. Reasoned requests for data access may be directed to the Research Lab at the Department of Psychosomatic Medicine and Psychotherapy, University of Leipzig (forschung.psom@medizin.uni-leipzig.de), which serves as the permanent data access committee. Requests will be reviewed by the data protection coordinator of the University of Leipzig as well as the local ethics committee prior to any transfer. In accordance with the EU General Data Protection Regulation, only de-identified data may be shared.

## Abbreviations

ACLED: Armed Conflict Location & Event Data
CTQ: Childhood Trauma Questionnaire
ERQ: Emotion Regulation Questionnaire
FOWARS: Fear of War Scale
KUSIV-3: Interpersonal Trust Short Scale
SD: Standard deviation
USUMA: Unabhängiger Service für Umfragen, Methoden und Analysen
VIF: Variance inflation factor
