# Supplementary material for: War-related and psychological predictors of experiential and physiological fear of war in Germany: findings from a nationally representative survey
Source: BMC Public Health. 2026 Sep 22;26:2649. doi: 10.1186/s12889-026-29548-7 (PMC13595654; doi:10.1186/s12889-026-29548-7)
Supplement: Supplementary file 1 [file 12889_2026_29548_MOESM1_ESM.docx]

**Table S1**

*Games–Howell post hoc comparisons of experiential and physiological fear of war across war-related salience categories*

|  | **Experiential fear of war** | | | **Physiological fear of war** | | |
| --- | --- | --- | --- | --- | --- | --- |
| **Comparison** | **Mean difference** | ***SE*** | ***p*** | **Mean difference** | ***SE*** | ***p*** |
| No specific war – Specific war | **-0.30** | 0.04 | < .001 | 0.06 | 0.05 | .68 |
| No specific war – Multiple wars | **-0.42** | 0.08 | < .001 | 0.03 | 0.10 | 1.00 |
| No specific war – Global war | **-0.58** | 0.08 | < .001 | **-0.71** | 0.10 | < .001 |
| No specific war – Nuclear war | **-0.81** | 0.15 | < .001 | **-0.98** | 0.19 | < .001 |
| Specific war – Multiple wars | -0.11 | 0.08 | .56 | -0.03 | 0.09 | 1.00 |
| Specific war – Global war | **-0.28** | 0.07 | < .001 | **-0.78** | 0.09 | < .001 |
| Specific war – Nuclear war | **-0.51** | 0.14 | .01 | **-1.04** | 0.19 | < .001 |
| Multiple wars – Global war | -0.16 | 0.10 | .45 | **-0.74** | 0.12 | < .001 |
| Multiple wars – Nuclear war | -0.40 | 0.16 | .10 | **-1.01** | 0.21 | < .001 |
| Global war – Nuclear war | -0.24 | 0.16 | .56 | -0.27 | 0.21 | .70 |

**Note.** Mean differences represent the difference between the first and second group listed. *SE* = standard error. Games–Howell post hoc tests were used to compare experiential and physiological fear of war across war-related salience categories.
